# Optimization of fortified sponge cake by nettle leaves and milk thistle seed powder using mixture design approach

**DOI:** 10.1002/fsn3.2041

**Published:** 2020-12-05

**Authors:** Fariba Ataei Nukabadi, Mohammad Hojjatoleslamy, Hajar Abbasi

**Affiliations:** ^1^ Department of food science and technology Shahrekord branch, Islamic Azad University Shahrekord Iran; ^2^ Department of food science and technology Korasgan branch, Islamic Azad University Isfahan Iran

**Keywords:** fortified sponge cake, milk thistle, mixture design, nettle

## Abstract

Powdered nettle leaf and milk thistle (MT) seed were added to the cake batter with certain percentages selected by the Design‐Expert v. 10 software (0–25, 25–0, 18.75–6.25, 6.25–18.75, and 12.5–12.5). Addition of nettle and MT seeds to the cake reduced the moisture content, volume, and springiness and increased hardness of the samples. 12.5% nettle‐12.5% MT seed had the least hardness and the highest amount of springiness and cohesiveness. The highest BI, WI, SI, L*, a*, and b* and the lowest Δ*E* were observed in 12.5% nettle‐12.5% MT seed and 25% MT seed samples, respectively. Antioxidant activity and antimicrobial properties were increased in all samples compared to the control sample, so that 6.25% nettle‐18.75% MT exhibited the highest antioxidant activity and antimicrobial properties. The highest levels of quercetin and silymarin were observed in 25% nettle and 25% MT seeds, respectively. In the sensory evaluation, 12.5% nettle‐12.5% MT seed took the best scores regarding flavor, texture, color, and overall acceptance. Based on the lowest hardness, 13.65% nettle‐11.34% MT seed was determined as optimized points by the software, which was equivalent to desirability of 0.72. The optimum sample contained 62.90 mg quercetin and 886.70 mg silymarin. According to the HPLC analysis results, consumption of 10 optimal cakes daily could theoretically decrease the blood sugar level, which requires further studies. The remaining amount of quercetin and silymarin in 2.5 g of nettle leaves and 2.5 g of MT seeds after heating was 11 and 19 mg, respectively. In other words, heat did not have much effect on the destruction of quercetin and silymarin.

## Graphical abstract

Proved that nettle leaf and milk thistle (MT) have therapeutic effect on human diseases and antioxidant activity. Highest BI, SI, L*, b*, and a* were found in sample containing 12.5% nettle and 12.5% MT seed. Optimum mixture had 0.023% *w*/*w* quercetin and 0.038% *w*/*w* silymarin.

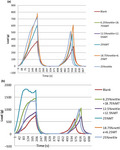

## 1 INTRODUCTION

Medicinal plants have many medicinal properties, and today, people tend to consume them. In addition, medicinal plants have many antioxidant and antimicrobial properties due to the presence of substances such as flavonoids and phenols, so that they can be properly used in the food industry.

Many of them can be substituted for chemical material as preservatives, antioxidants, flavorings, and pigments in products, and they have been introduced to the customer as a functional food; therefore, they have a good market. The cereal industry is almost the largest and important industry among various food industries worldwide. One of the problems with cereal products is the lack of fiber. The use of various herbs in the food industry can provide fiber to the product (Ataei & Hojjatoleslamy, 2017). Consumption of a combination of many herbs may be harmful to humans. Nettle (*Urtica dioica* L.) and milk thistle (*Silybum marianum* L.) are instances of herbs containing fiber, and which can be used in cereal products, causing no problem to human health if they are used together (Ahmed KK & Parsuraman, 2014; Khalili et al., 2017).

Nettle is a perennial plant naturally growing in regions with temperate and tropical climate across the world. Nettle has many medicinal properties and effective in treatments such as anemia, arthritis, and hay fever. An external use reported for this plant is the treatment of skin disease, gout, sciatica, neuralgia, hemorrhoids, hair problems and so forth. Additionally, it has been famous for its effect on lowering the fasting blood sugar together (Ahmed KK & Parsuraman, 2014).

Nettle is rich in ash, protein, vitamins A and C, calcium, iron, sodium, fatty acid, dietary fiber and carbohydrate (Adhikari et al., 2016). It also contains chemicals such as flavonoids, agglutins, lignans, carotenoids, phenolic compounds, and terpenoids. Flavonoids are compounds known to be helpful in improving diabetic indices (Song et al., 2005). Kaempferol, quercetin, isorhamnetin, isoquercitrin, astragalin, rutin, and their 3‑rutinosides and 3‑glycosides are the flavonoids found in nettle. Quercetin, mainly present in the plant leaves, is a popular natural antioxidant (Abenavoli et al., 2018).

Milk thistle (MT) is an annual or biennial flowering plant growing all over the world. MT is recommended for the treatment of varicose veins, menstrual disorders, splenic congestion, kidney, and liver in the early 19th century (Abenavoli et al., 2018).

Silymarin, extracted from MT seeds and then dried in standard conditions, contains mostly flavonolignans (up to 80% w/w) in addition to polymeric and oxidized polyphenolic compounds. Silymarin is also reported to have positive effects on the blood sugar control and reduction (Luminita et al., 2016). Silymarin is composed mainly of silybinin, silychristin, silydianin, and isosilybinin with approximate percentages of 60, 20, 10 and 5%, respectively. Furthermore, diastereoisomers of the mentioned compounds, including silybinin A, silybinin B, isosilybin A, isosilybin B are found in addition to taxifolin. The use of nettle and MT in cereal products such as cakes and the resistance of silymarin and quercetin to the heat process can produce functional foods that have medicinal properties, texture quality and consumer acceptance, and lead to an increase in human health (Tayoub et al., 2018).

Ataei and Hojjatoleslamy (2017) evaluated the amount of oleuropein remaining in the cake.

They reported that oleuropein remained more in the olive leaf powder than its extract did. Therefore, in this study, nettle leaves and MT seeds were added to the cake in powder form. Moreover, the amount of silymarin and quercetin, which is effective in reducing blood sugar, was evaluated (Ataei & Hojjatoleslamy, 2017).

In addition, the mixture design method was used, which contributes to the reduction of the number of treatments and repeat tests, so that results are obtained faster.

## 2 MATERIALS AND METHODS

The following ingredients were used to make sponge cakes: cake flour (Golard Co., Isfahan, Iran), liquid oil (Margarine Co., Tehran, Iran), sugar (Isfahan Sugar Co., Isfahan, Iran), dry milk powder (Palood Parsian Food Industries Co., Neyshabour, Iran), baking powder (Hermin Co. Shahriyar, Iran), whey protein (Pegah Co., Golpayegan, Iran), vanilla powder (Polar Bear, A. M Food Chem. Co., China) and fresh eggs (Local market), and water. Nettle leaves and MT seeds were provided by the Seed and Plant Improvement Institute (Karaj, Iran).

### 2.1 Sponge cake preparation

We prepared the sponge cake according to the recipe introduced by the authors in an earlier study with some modifications. Table 1 shows the ingredients based on 100 g of flour. Nettle leaf and MT seed powder was added to the formulation in the last step of batter preparation. After cooling, the baked cakes were packed in poly propylene bags at room temperature and kept for future physicochemical and sensory evaluation analyses (Ataei & Hojjatoleslamy, 2017).

**TABLE 1:** Formulation of sponge cakes (g)

Nettle powder	MT seed powder	Cake flour	Sucrose	Whole egg	Oil	Baking powder	Vanillin	Whey protein powder	Dry milk	Water
0	25	100	72	72	57	1.34	0.5	4	2	25
25	0	100	72	72	57	1.34	0.5	4	2	25
18.75	6.25	100	72	72	57	1.34	0.5	4	2	25
0	25	100	72	72	57	1.34	0.5	4	2	25
6.25	18.75	100	72	72	57	1.34	0.5	4	2	25
12.5	12.5	100	72	72	57	1.34	0.5	4	2	25
12.5	12.5	100	72	72	57	1.34	0.5	4	2	25
25	0	100	72	72	57	1.34	0.5	4	2	25
0	0	100	72	72	57	1.34	0.5	4	2	25

### 2.2 Optimization of mixing ratio

The mixture Design of Experiment method was used to find the optimum mixture ratio of the nettle‐MT powder (*p* < .05). The Design‐Expert (V. 10) software, Stat‐Ease Inc., USA, was used in this research.

Nettle leaf and MT seed were assumed to be independent variables with a total proportion of 25% in a 100‐g sponge cake. The software then suggested the amount of nettle and MT seed for different treatments (Table 1). The best model expressing the behavior of the treatments was chosen according to the obtained p‐value. Moreover, more parameters, including R^2^ and Adjusted R^2^, were studied where the lack of fit test was not significant. Finally, the response and the dependent variable were considered desirable based on the independent variables as: (Mohammadi et al., 2019).

(1) $$\left[D={\left(d_{1}\times d_{2}\times \ldots \times d_{n}\right)}^{1/n}={\left(\prod_{i=1}^{n}d_{i}\right)}^{1/n}\right]$$

where; D, is total desirability, d, is desirability of each response, and n, is response number.

### 2.3 Chemical properties of sponge cake

Chemical properties of the cake samples were measured using AACC approved methods as: moisture content, crude protein, ash content and crude fat by AACC 46–40, AACC 46‐11A, AACC 08–01, and AACC 30–10, respectively. According to the method AACC 46‐11A, the crude protein content was assessed using the nitrogen conversion factor of 6.25 (Ataei & Hojjatoleslamy, 2017).

### 2.4 Physical properties of sponge cake

The cake volume and density were measured according to Prokopov et al. using rapeseed displacement (Prokopov et al., 2015). Samples with dimensions of 20 × 20 × 20 mm were taken from the midsection of the cakes on the first and after thirty days of storage for the texture profile analysis (TPA) using a texture analyzer (Brookfield Engineering, Middle borough CT_3_, USA). With a programmed double cycle, the texture profile was determined. Various test conditions (up to 50% deformation; crosshead speed of 15 mm/s) were applied. A TA‐25/1000 probe was used for hardness, cohesiveness and springiness determination. Moreover, a TA‐41 probe was employed to perform the punching test up to 10 mm deformation (Ataei & Hojjatoleslamy, 2017).

Springiness quantifies the elasticity of the cake by measuring the distance recovered between the first and second compressions. Cohesiveness indicates the extent at which the product texture resists to the second deformation relative to the first one (Salehi et al., 2016).

The Hunter Lab system was used to determine the color characteristics of the crumb of cake samples using a ColorFlex Ez, USA colorimeter. The L*, a* and b* values were determined. Where the L* value indicates the lightness from dark (0) to light (100), the a* value indicates the degree of the green to red color, the higher positive the value the more reddish. The value b* represents the yellow to blue color, the higher positive the value the more yellow. Δ*E*, which shows total color difference from the reference color, was obtained from Equation 2 (Hafez, 2012).

(2) $$\Delta E=\sqrt{{\left(L_{0}‐L^{*}\right)}^{2}+{\left(a_{0}‐a^{*}\right)}^{2}+{\left(b_{0}‐b^{*}\right)}^{2}}$$

The Saturation Index, also known as chroma, indicating the degree of the color being proportional to the intensity of the color, was calculated by Equation 3 as follows: (Saricoban & Yilmaz, 2010).

(3) $$\text{SI}=\sqrt{a{*}^{2}+b{*}^{2}}$$

Hue is generally demonstrated by a single angular number in the range of 0°–360°, where 0°= bluish‐red, 90°= yellow, 180°= green and 270° = blue (Saricoban & Yilmaz, 2010).

(4) $$H=\arctan\left(\frac{b*}{a*}\right)$$

The Browning Index (BI) shows the purity of brown color as an important parameter in food processing practices in which enzymatic and non‐enzymatic browning may occur (Sedaghat Boroujeni & Hojjatoleslamy, 2018).

(5) $$BI=\frac{\left[100\left(x‐0.31\right)\right]}{\left(0.17\right)}$$

where; $x=\left(a*+1.75L*\right)/\left(5.645L*+a*‐0.3012b*\right)$.

### 2.5 Quercetin and Silymarin measurement

To evaluate the quercetin and silymarin retention in baked cakes, the amount of quercetin and silymarin was measured. The measurement was performed according to the method proposed by Haghi and Hatami (2010) and Bourgeois et al., 2016), using the HPLC technique.

The HPLC system (Azura, Knauer Co) with a k‐1001 pump and a UV‐Visible detector k‐260 was used for this purpose. The Teknokrom C18 column was chosen for the analysis, and the UV‐visible detector was set at 370 nm and 150 nm for quercetin and silymarin, respectively. The mobile phase to measure quercetin was acetonitrile (7%) (A) and phosphoric acid (B). Injection volume was 20 μl, and the column temperature was ambient temperature. The elution protocols applied for quercetin were: 0–9 min, 75% B; 9–19 min, 75%–25% B; and 19–24 min, 25% B (Bourgeois et al., 2016).

A ternary mixture elution system of methanol (A), water (B), and 1% aqueous acetic acid solution (C) was used at a flow rate of 1.0 ml/min to measure silymarin. The linear gradient was: (0 min) 30% A‐50% B‐20% C, (5 min) 35% A‐45% B‐20% C, (20 min) 35% A‐45% B‐20% C, (25 min) 45% A‐35% B‐20% C, (60 min) 45% A‐35% B‐20% C. Injection volume was 20 μl throughout the experiment at ambient temperature. Peak identification was based on the literature data, relative retention times, spectra matching and available standards. Silymarin and quercetin contents were obtained from Equation 6 (Cai et al., 2009).

(6) $$\%\left(\text{Silymarin}‐\text{Quercetin}\right)=\frac{\left(A_{u}\times C_{s}D_{f}\right)}{\left(A_{s}\times M\times 1000\right)}$$

### 2.6 Microbial load determination

The microbial load of cake samples was determined according to the International Commission on Microbiological Specification of Foods (ICMSF, 2009). For this purpose, a unit weight of a cake sample was taken and mixed with 9 ml of sterile distilled water in a test tube. It was then serially diluted until the desirable dilutions were obtained. A volume of 0.01 ml aliquot from each diluent was aseptically transferred into different previously autoclaved plates poured with already sterilized molten media. The plates were then incubated at 28°C for 72 hr.

The plates in the incubator were daily checked for growth. The number of grown colonies on each plate was counted using a colony counter. The number of colonies was reported in term of the number of the colony forming unit (CFU).

### 2.7 Determination of antioxidant activity

Free radical scavenging activity, DPPH radical elimination, was determined according to the method proposed by Teh et al. (2014), with some modifications. A 1 ml volume of the extract was mixed with 4 ml of 25 mM DPPH methanolic solution. The obtained solution was stirred and maintained in the dark for 30 min before being measured by a spectrophotometer (Jeneway, England) at 515 nm, against a blank cell containing only methanol. Results were expressed as the inhibition percent of DPPH according to the following equation (Equation 7):

(7) $$\%\;\text{inhibition}\;\text{of}\;\text{DPPH}=100\times \frac{\left(\text{Absorbance}\;\text{control}‐\text{Absorbance}\;\text{sample}\right)}{\text{Absorbance}\;\text{control}}$$

where absorbance control is the absorbance of DPPH solution without extract.

### 2.8 Sensory evaluation

The hedonic scale as a unique and commonly used scale to measure food product liking and preference yields reliable results. Hence, a 5‐point hedonic scale, according to the method employed by the authors in a previous study was conducted to determine the degree of overall liking of the sponge cakes (Ataei & Hojjatoleslamy, 2017). A total of 20 semi‐trained panelists were selected, each panelist received five cake slices, cut from the midsection of the cakes maintained at ambient temperature, and was asked to score each sample based on the degree of liking on a five‐point hedonic scale (one: dislike very much, two: dislike, three: neither dislike nor like, four: like, five: like very much). The panelists received samples.

## 3 RESULTS AND DISCUSSION

### 3.1 Physicochemical characteristics of sponge cake

Table 2 presents the proximate chemical composition including moisture content, fat content and protein content of the cake samples. The moisture content on the first and thirtieth days of storage ranged from 13.5% to 19.32% and from 8.06% to 16.5, respectively. The cake sample containing 12.5% nettle and 12.5% MT seed had the highest moisture content on the first and thirtieth days of storage. High moisture content of this sample could be attributed to the high water binding capacity of the leaves' powder. According to Table 3, the quadratic model could indicate the moisture content as the function of the added plant powder in the first‐day well, while the quartic model was the best model representing it on the thirtieth day. Regarding the highest moisture content in the 12.5% nettle‐12.5% MT seed cake on the first day, the highest moisture content among the samples after 30 days of storage was expected for this treatment. However, the moisture content of all samples was reduced during storage time due to the occurrence of staling during shelf life. Addition of nettle leaf and MT seed resulted in a reduction in the moisture content of the incorporated cake samples compared to the control sample. Nettle and MT seeds could have transferred water from the protein network to the starch network by altering the structure of starch. However, the equal amount of the two powders in combination increased the moisture retention compared to other samples with different combinations. Corresponding to our results, Hafez (2012) for cake with Marjoram reported the lower moisture content at the end of storage time because of retrogradation. These results were also consistent with the results obtained by Ataei and Hojjatoleslamy (2017) in which the olive leave powder was added to the sponge cake. They reported weakened gluten network and decreased moisture content by adding olive leaves to the cake. Tsong‐Ming Lu et al. (2010) reported that addition of green tea to the sponge cake had no significant effect on the moisture content of the cake.

**TABLE 2:** Physicochemical characteristics of sponge cake prepared with nettle and MT seed powder

Chemical characteristics										
Sample	Nettle (g)	MT seed (g)	Moisture% (day 1)	Moisture% (day 30)	Fat (%db)	Protein (%db)	Ash (%db)	Quercetin (mg)	Silymarin (mg)	Inhibition of DPPH (%)
1	12.5	12.5	19.32	16.5	38.2	12.83	1.14	57.7	950	42
2	12.5	12.5	19	16.04	37.75	12	1.12	57	900	40
3	0	25	14	8.43	39.2	14.2	1.08	0	2,025	27
4	18.75	6.25	18.14	15.48	37.04	12.5	1.2	86.25	575	40.02
5	25	0	16.48	11.02	36.32	11.27	1.22	115	0	18
6	0	25	13.5	8.06	38.73	13.5	1.06	0	1,552.5	25
7	25	0	16	10.42	36	10.5	1.3	116	0	20
8	6.25	18.75	17.51	15.9	38.5	13.52	1.1	28.75	1,775	47
9	0	0	21	13	34.58	11.04	1.23	0	0	0

Physical characteristics										
Antimicrobial Cfu/g	Volume (cm^3^)	Density (g/cm^3^)	Hardness (g) (Day1)	Hardness (g) (Day 30)	Cohesiveness (Day 1)	Cohesiveness (Day 30)	Paunch (g) (Day 1)	Paunch (g) (Day 30)	Springiness (mm) (Day 1)	Springiness (mm) (Day 30)
2.2	82.54	0.55	467	896	0.45	0.34	83.5	219	7.65	6.01
2.1	83.42	0.54	446	840	0.45	0.34	86	205	7.52	6.14
1.1	67.19	0.63	665	1,834	0.35	0.16	133.5	437	6.31	4.01
2.2	77.7	0.61	480	1,348	0.43	0.28	112	284.5	7.32	5.43
1	71.33	0.64	660	1,385	0.4	0.22	124.5	378	6.78	4.78
1.1	67.02	0.71	690	1,734	0.34	0.15	147.4	449	6.28	4.12
1	72.33	0.63	620	1,324	0.4	0.21	123	420	5.67	4.55
2.3	75.46	0.64	578	1,453	0.38	0.27	124.5	355	6.78	5.02
0.5	84.8	0.53	373	750	0.45	0.34	109.6	205	8.13	7.57

**TABLE 3:** Analysis of predicted model equation for the quality characteristics of cake prepared with nettle and MT seed

Response	Model	*F*‐value	Prob < *F*	*R* ^2^	Adj *R*‐squared	Equation on terms of pseudocomponent
Hardness (day1)	Quadratic	33.41	.0013	.93	0.90	632.92A + 688.03B − 774.59AB
Springiness (day1)	Quadratic	7.51	.0312	.75	0.65	6.27A + 6.22B + 4.98AB
Paunch (day1)	Quartic	24.93	.0123	.97	0.93	123.75A + 140.45B − 189.40AB
Cohesiveness (day1)	Quartic	185.25	.0006	.99	0.99	0.4A + 0.34B + 0.31AB
Moisture (day1)	Quadratic	142.53	<.0001	.98	0.97	16.15A + 13.80B + 16.20AB
Hardness (day30)	Quartic	75.81	.0024	.99	0.97	1,354.5A + 1784B − 2,805AB
Springiness (day30)	Quartic	81.85	.0022	.99	0.97	4.66A + 4.06B + 6.84AB
Paunch (day30)	Quartic	45.74	.0051	.98	0.96	399A + 443B − 836AB
Cohesiveness (day30)	Quartic	284.16	.0003	.99	0.99	0.22A + 0.15B + 0.62AB
Moisture (day30)	Quartic	192.63	.0006	.99	0.99	10.72A + 8.24B + 27.15AB
Fat	Linear	107.49	<.0001	.94	0.93	36.31A + 39.13B
Protein	Linear	35.15	.001	.85	0.82	11.11A + 13.97B
Ash	Linear	46.86	.0005	.88	0.86	1.25A + 1.06B
% inhibition of DPPH	Quartic	107.52	.0014	.99	0.98	19A + 26B + 74AB
Density	Linear	7.16	.0367	.66	0.52	0.65A + 0.55B
volume	Quartic	231.35	.0005	.99	0.99	71.83A + 67.11B + 54.05AB
a*	Quadratic	20,349.4	<.0001	.99	0.99	−0.050A + 1.75B − 7.30AB
b*	Quartic	931.26	<.0001	.99	0.99	4.99A + 9.93B + 2.20AB
L*	Quartic	2,874.57	<.0001	.99	0.99	11.73A + 30.05B − 14.68AB
Δ*E*	Quartic	2,846.93	<.0001	.99	0.99	30.41A + 11.64B + 13.13AB
SI	Quartic	931.08	<.0001	.99	0.99	4.99A + 10.08B + 2.10AB
*H*	Quartic	3,436.53	<.0001	.99	0.99	−89.42A + 77.89B − 309.16AB
BI	Quadratic	205.40	<.0001	.98	0.98	77.30A + 82.18B + 7.70AB
WI	Quartic	2,743.43	<.0001	.99	0.99	11.09A + 29.32B − 14.54AB
Quercetin	Linear	51.71	.0005	.95	0.93	0.12A + 2.549B − 0.12AB
Silymarin	Linear	87.70	<.0001	.93	0.92	1.754A + 7.602B
Flavor	Quadratic	10.66	.0157	.81	0.73	2.41A + 1.52B + 7.22AB
Odor	Linear	8	.03	.57	0.50	3.08A + 4.42B
Texture	Quadratic	14.13	.0088	.84	0.78	2.90A + 2.01B + 5.02AB
Color	Cubic	644.67	<.0001	.99	0.99	1.01A^2^ + 4.01B^2^
Over all	Quadratic	10.68	.0157	.81	0.73	1.98A + 2.43B + 6.12AB
Antimicrobial	Quartic	400.5	.0002	.99	0.99	1A + 1.10B + 4.40AB

Note

*A*: Nettle, *B*: MT Seed.

Table 2 indicates the volume and density measured for the cake samples prepared based on the results of the mixture design of experiment. The volume and density of the cake samples on the first day ranged from 67.02 to 83.42 cm^3^ and from 0.54 to 0.71 g/cm^3^, respectively. The quartic model properly fitted to the experimentally measured volume results, while the linear model had the best fitting to those of density. According to the results presented in Table 2, addition of the nettle leaf and MT seed powders caused to decrease the cake volume and to increase its density, compared to the control sample. The cake volume is generally influenced by the characteristics of the fibers added, via both the nature and amount. Fiber addition can disturb the gluten network, and eventually the gluten protein is diluted. A weak gluten network might result in letting gases such as carbon dioxide and water vapor to escape from the cake pores and consequently decrease the cakes volume (Kim et al., 2012; Tsong‐Ming lu et al., 2010). It appears that it occurred more considerably in the sample containing the 25% MT, so that it had the lowest volume, while the 12.5% nettle‐12.5% MT seed had the highest volume among the cake samples. These findings were consistent with those reported by Aydogdu et al. (2018) in one study of the rheological properties of cake batter and quality of the final product as affected by addition of different fibers. It was stated that a significant correlation was found between the consistency index and the specific volume. As the batter consistency index was increased, cakes with lower specific volume were obtained. The type of fiber is highly important. The consistency index of the batter containing lemon and apple was higher than that with oat and pea. Particularly, addition of lemon fiber resulted in the highest consistency index of batter and the lowest specific volume of cakes. Gómez et al. (2003) reported that the lowest volume of bread was obtained, when the coffee fiber with higher soluble fiber content was added to bread. Furthermore, higher elastic and viscous modulus values inhibited cake development, leading to lower volume.

Table 2 presents the results of protein, ash and fat content measurement. A linear model was selected to fit fat, protein and ash content results. MT is rich in protein and fat. It contains 25.25% protein and 29% fat depending on the variety, type of cultivation and climate (El‐haak et al., 2015). Nettle is also rich in minerals such as sodium, iron and various vitamins (Adhikari et al., 2016). Accordingly, the 25% MT seed sample was demonstrated to have the highest amount of fat and protein. However, the lowest levels were measured in the 25% nettle sample. Moreover, the highest ash content was measured in the 25% nettle sample. It was attributed to the high ash content of the nettle leaves.

**FIGURE 1:**
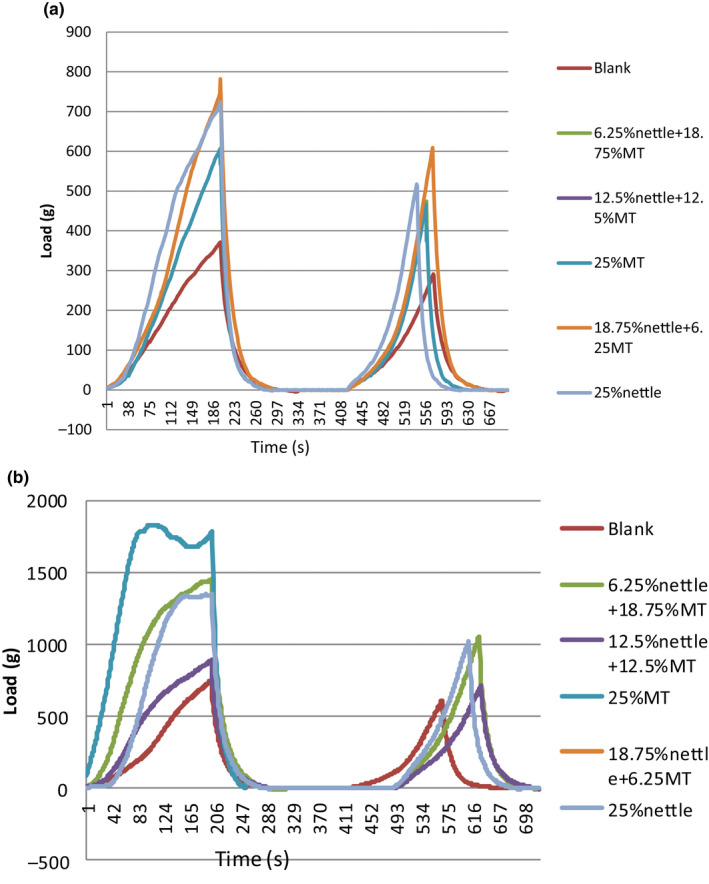
Texture profile analysis (TPA) of different samples (a) day1 and (b) day30

**FIGURE 2:**
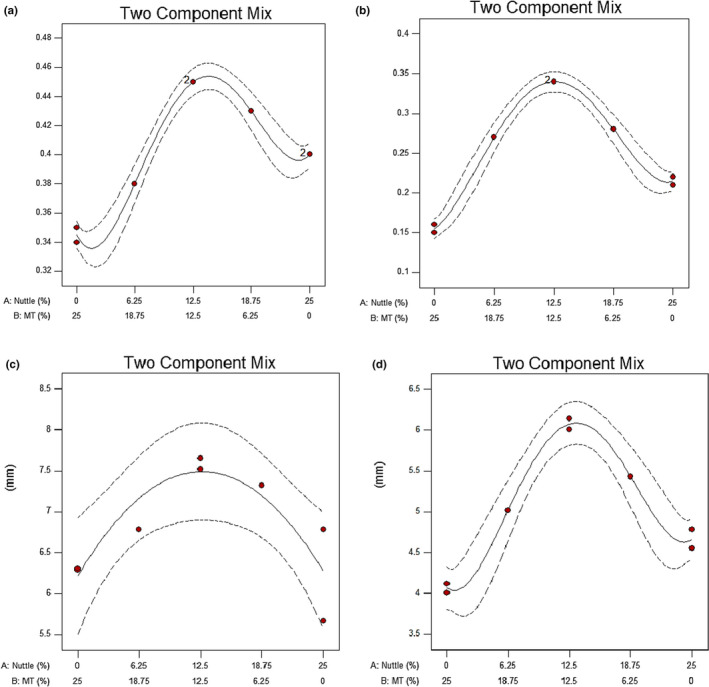
Diagram of mixture design (a) cohesiveness (day1), (b) cohesiveness (day30) (c) springiness (day1), and (d) springiness (day30)

Table 2 and Figure 1 represent the results of the texture analysis of the cake samples. The Adj. R‐squared and R^2^ values indicate the goodness of fit of the model to the experimental data. The lowest and highest hardness values were measured for the samples containing 12.5% nettle‐12.5% MT seed and 25% MT seed, respectively (Figure 2).

Hardness is a combination of the crumb and crust strength under compression. It is expressed as the maximum point in the force versus deformation diagram (Yarahmadi et al., 2019). In this test, contrary to the punch test, the hardness of the probe does not lead to rupture of the cake tissue. In addition, the resistance of the cake is directly associated with the density and indirectly with the volume of the cake, so that as the density increases, the resistance of the cake increases, and therefore, the hardness increases (Tsong‐Ming lu et al., 2010). In this study, the results of volume and density tests are consistent with the results of hardness tests. The cake sample containing 12.5% nettle and 12.5% MT seed had the highest volume and the lowest hardness, and the 25% MT seed sample had the lowest volume and the highest hardness.

**FIGURE 3:**
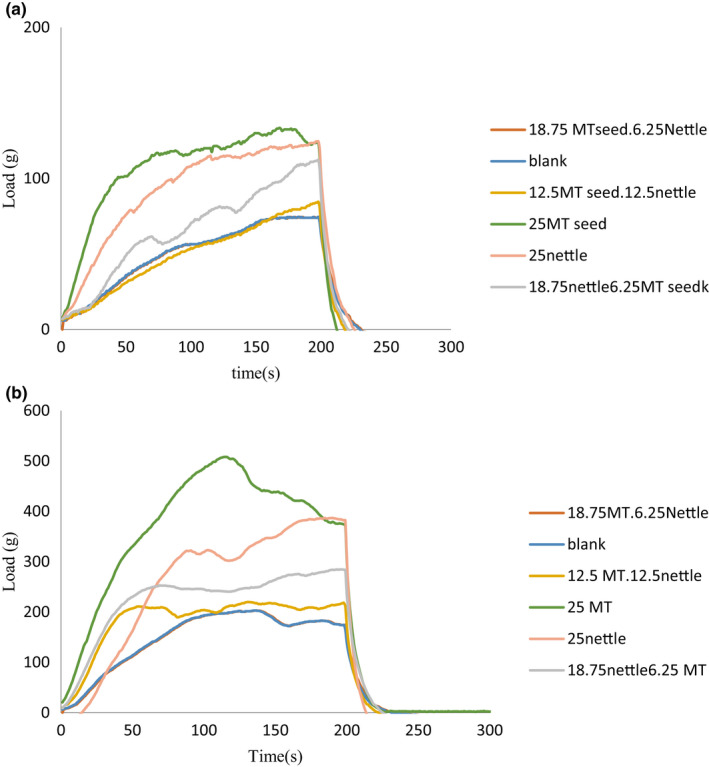
Hardness of punch test in different samples (a) day 1 and (b) day 30

The results of the texture analysis correspond to those of the moisture content measurement. In fact, an explicit reverse relationship could be observed between the moisture content of the samples and their texture hardness. The 25% MT seed sample had the lowest moisture content and the highest hardness, while the 12.5% nettle‐12.5% MT seed sample revealed the highest moisture content and the lowest hardness. Furthermore, a predictable reduction of the moisture content of the samples occurred during storage time, being concurrent with the hardness increase of the samples. Figure 3

These results correspond to those obtained by Seo et al. (2010) in the study of the sponge cake containing turmeric. Addition of turmeric to the cake increased the cake hardness, gumminess, and chewiness, and thus the softness of the cake was decreased due to its effect on the gluten network. By addition of 129.5% sugar, 0.5% turmeric powder, and 10.0% oil, linear models were selected for the hardness, gumminess, and chewiness (Seo et al., 2010).

The quadratic model and the quartic model were the best model fitting to the measured springiness on days 1 and 30, respectively. The quartic model was also selected as the most appropriate model to fit to cohesiveness results. Both cohesiveness and springiness values of the 12.5% nettle‐12.5% MT seed sample were higher than all other samples. It implies that the use of the nettle leaf and MT seed powder in the cake weakened the gluten network and as a result reduced the cohesiveness and springiness of the samples. However, the most significant decreased cohesiveness and springiness were observed in the 25% MT seed sample.

Cohesiveness depends on the internal resistance of the food structure. Decreased cohesiveness can be owing to the weakening of the gluten network. The gluten network is a combination of glutenin and gliadin. It is formed when flour and water are mixed together during stirring. In fact, the gluten network is a sticky and reversible mass containing other components such as starch and gas bubbles, thereby providing the basis for the formation of the main structure of the cake. Addition of some materials can weaken the network and reduce the strength of the structure. In addition, during the storage period of the cake, this network gradually is weakened, the internal strength of the cake structure is decreased, and consequently the cohesiveness and springiness of the cake are decreased (Kim et al., 2012).

In this study, the results revealed that the weakening of the gluten network and eventually reduction of cohesiveness and springiness occurred largely when nettle and MT seed were simultaneously added to the cake compared to the control. However, addition of either nettle or MT seed alone led to further disruption of the gluten network and further reduction of the cake cohesiveness and springiness. The results were in agreement with the findings obtained by Tsong‐Ming lu et al., 2010) for the cakes added with the green tea powder. They replaced different percentages (0%, 10%, 20%, and 30%) of the cake flour by the green tea powder and reported that the viscosity of the cake batter was higher than that of the control sample, but the cake volumes exhibited a reverse trend. It was attributed to the increased replacement of flour with cellulose and the weakened gluten matrix. Thus, the cohesiveness and springiness were decreased with addition of the green tea powder.

Kim et al. (2012) reported that hardness and gumminess were reduced, and cohesiveness and springiness were increased with addition of the cactus *Opuntia humifusa* powder. This was due to the mucilaginous properties of the cactus pectin. Gums displaying viscous properties in the solution state were added to cake batters in order to increase the moisture retention during baking. Pectin preserves the moisture, increases the volume, springiness and cohesiveness of cake, and decreases its hardness.

### 3.2 Antioxidant activity

Antioxidants serve as preventatives from the destructive and detrimental influence of free radicals, also known as oxidants, on the cells and the resulting diseases. Therefore, assessment of antioxidant and free radical scavenging characteristics of different natural products and additives has been the aim of many research studies. Polyphenols, including phenolic acids, and flavonoids are the major and most popular compounds with antioxidant properties (Kamkar & Khodabakhshiyan, 2017; Viktorova et al., 2019).

Nettle and MT are known to have antioxidant properties owing to their high phenolic and flavonoid content.One of the most important flavonoids contained in MT is silymarin, which has high antioxidant activity (Viktorova et al., 2019).

According to the literature, antiradical properties and inhibitory potential of nettle are greater than those of many synthetic antioxidants (Zeipina et al., 2015). Antioxidant activity of nettle leaves was reported to range from 17.31% to 80.77%. Zeipina et al. attributed this variation to the fertility of soil, the clone and the plant age. (Zeipina et al., 2015). Saa (2017) studied the antioxidant properties of silymarin and reported that silymarin had higher antioxidant properties than BHT had. As different concentrations of silymarin were added to the sunflower oil, the antioxidant activity of the sunflower oil was increased compared to BHT. Table 2 presents the results of the antioxidant activity measurement. The quartic model was selected by the software to fit the data. According to our findings, the highest and lowest DPPH scavenging elimination was observed in the 6.25% nettle‐18.75% MT seed sample and the 25% nettle cake. It appears that the combination of nettle and MT seeds used in the cake could exhibit higher antioxidant effect than when used alone.

### 3.3 Antimicrobial activity

Table 2 shows the microbial test results. The quartic model is reported as the proposed model in the table. The lowest amount of mold and yeast was counted in the 6.25% nettle‐18.75% MT seed sample, owing to their highest content of phenolics and flavonoids, and antioxidant activity. Studies have indicated a direct relationship between antioxidant and antimicrobial properties of materials. Studies have demonstrated a direct relationship between antioxidants and antimicrobial properties of substances, so that as the amount of antioxidants in food increases so do antimicrobial properties (Fazelinasab et al., 2017). Both nettle and MT have antimicrobial and antioxidant properties owing to their phenolic and flavonoids content (Hasanloo et al., 2014; Moradi & Amini, 2017). The results of this study showed that the growth of mold in all treatments was lower than that of the control sample. It was due to the presence of nettle and MT seed in the formulations.

### 3.4 Quercetin and Silymarin content

**FIGURE 4:**
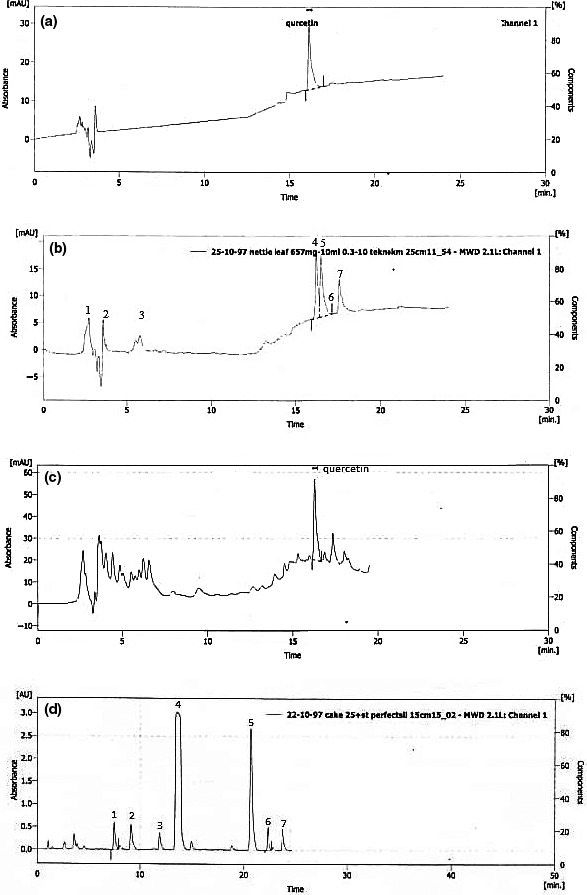
Chromatograms of Quercetin and silymarin. (a) Chromatograms of standard solutions for quercetin, (b) Chromatograms of standard solutions for silymarin, (c) Chromatograms of quercetin for cake, and (d) Chromatograms of silymarin for cake. 1:Taxifolin, 2:Silychristin, 3:Silydianin, 4:SilybininA, 5:SilybininB, 6:IsosilybininA, and 7:IsosilybininB

Table 2 and Figure 4 present the HPLC test results. Quercetin and silymarin are the main compounds measured in nettle and MT, respectively. The quercetin content ranged from 0 to 116mg, and the silymarin content ranged from 0 to 2,025 mg in the cake samples. The linear model was selected as the best model fitting to experimentally measured quercetin and silymarin values. As nettle and MT seed were increased, the quercetin and silymarin content was increased in the samples.

### 3.5 Color measurements

Table 4 presents the results of color measurements in different cake samples. The quartic model was demonstrated to be the best model fitting to ΔE, L*, b*, SI, a* and WI values, while the quadratic model exhibited the best fit to measured BI values.

**TABLE 4:** Color characteristics and sensory evaluation of sponge cakes

Nettle (%)	MT seed (%)	a*	b*	L*	∆E	SI	H	BI	WI	Texture	Flavor	Color	Odor	Overall liking
12.5	12.5	−0.98	8.02	17.44	24.09	8.07	−83.03	81.89	17.04	4	4	3	4	4
12.5	12.5	−0.97	8	17	24.52	8.05	−83.08	81.89	16.6	4	4	3	3	4
0	25	1.76	9.96	30.09	11.58	10.11	79.96	82.42	29.36	2	2	4	5	3
18.75	6.25	−2.04	5.34	12.31	29.85	5.71	−69.03	79.63	12.12	3	3	3	4	3
25	0	−2.04	5.1	11.78	30.33	5.1	−89.55	77.45	11.63	3	2	1	3	2
0	25	1.74	9.9	30	11.69	10.05	75.82	82	29.28	2	1	4	4	2
25	0	−2.21	4.88	11.69	30.49	4.88	−89.29	77.24	11.55	3	3	1	3	2
6.25	18.75	−0.36	9.22	20.29	20.99	9.22	−87.76	82.14	19.75	3	3	3	4	3
0	0	1.62	18.90	48.43	0	18.96	85.09	49.96	45.06	4	4	4	5	5

Samples containing 25% MT seed had the highest a*, b*, L*, WI, SI, BI and H values and the lowest color difference (Δ*E*) due to similarity of the seed powder color to the wheat flour used in the control sample. A close relationship was found between the color characteristics of the color of plant pigments added and the color of the final products. The distance between the color of the treatments and that of the control sample could be attributed to the oxidation and caramelization of pigments and phenolics during baking. These findings agreed with those reported by Nikmaram et al. in which they reported a darker color for the sesame added corn extrudates (Nikmaram et al., 2015). The MT seed color was yellow to brown, thus the cake containing only the MT seed powder had the highest b* and L* value. Moreover, the high value of a* was due to lack of green color pigments in the seed powder. However, regarding the green nettle leaf, as the percent of the nettle leaf was increased in the formulation, the a* and b* values were decreased. The cakes containing the nettle powder had a lower L* value than the other treatments. It was due to the darker color of nettle than the MT seed powder and the wheat flour.

### 3.6 Sensory evaluation

Table 4 presents the results of the sensory evaluation performed based on the 5‐point hedonic scale. Flavor, texture, and overall acceptance values were fitted by a quadratic model, while odor and color were predicted by linear and cubic models in the best way, respectively. Among all treatments, the cake with 12.5% nettle leaf and 12.5% MT seed gained the highest overall acceptance score.

### 3.7 Optimization

**FIGURE 5:**
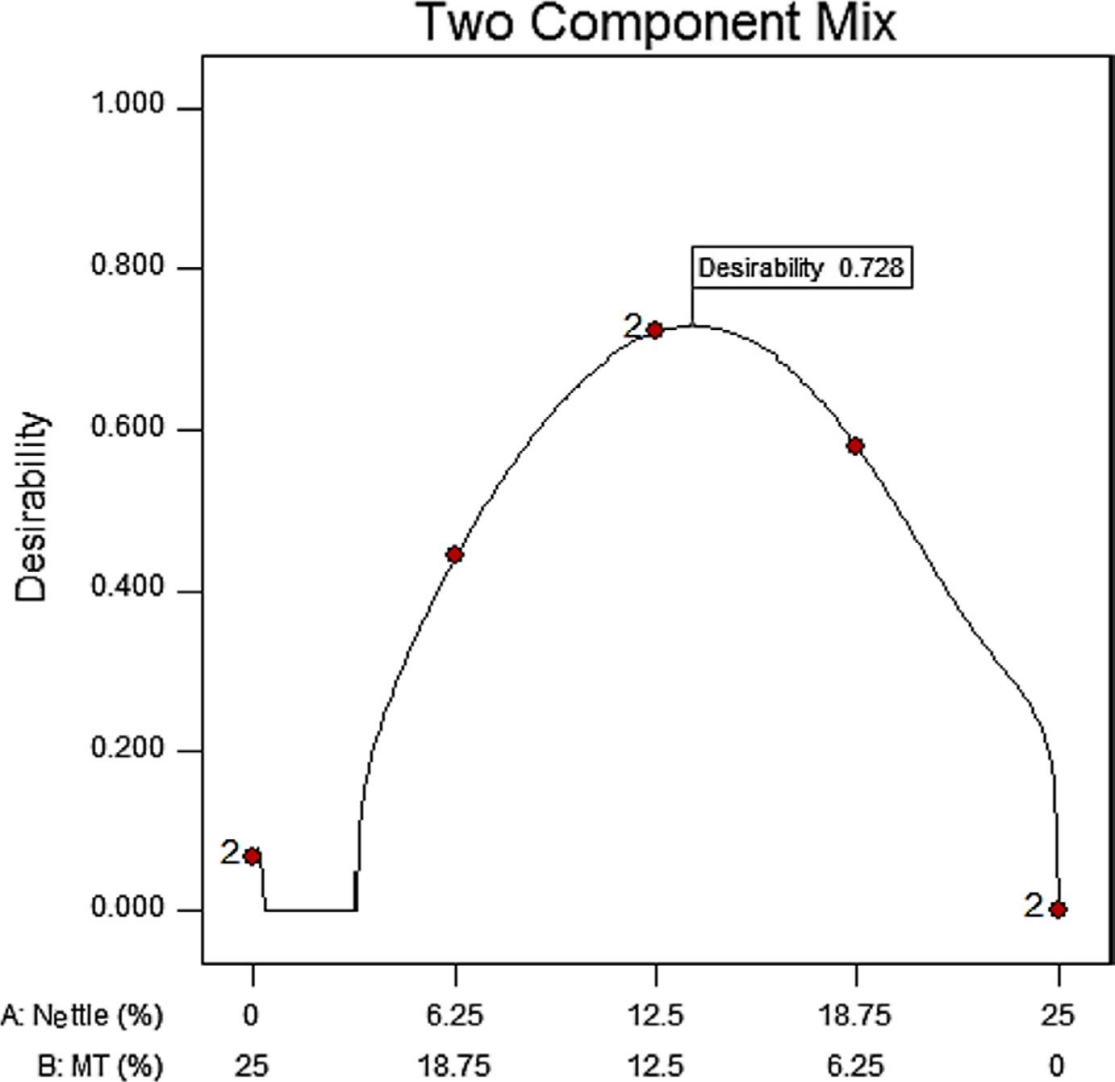
Desirability of optimized points

The optimized combination of the nettle leaf powder and the MT seed powder to be used in the cake was determined by the Design‐Expert software. Independent variables were assumed to be different amounts of nettle and MT seed, up to 25%, according to the minimum hardness value and the highest sensory test scores, and then optimization was performed by the software. According to Figure 5, the combination of 13.65% nettle and 11.34% MT seed powder was determined as the optimized point, which was equivalent to the desirability of 0.72 and contained 62.90 mg quercetin and 886.70 mg silymarin (Table 5). The chemical and physical tests were then performed for the optimum sample (Table 6). Studies have revealed that daily consumption of 200 mg of nettle and 200 mg of silymarin is effective in lowering the blood sugar in individuals with type 2 diabetes (Khalili et al., 2017). The results of the HPLC test that approximately 10 optimal cakes contained an adequate amount of quercetin and silymarin, which can theoretically decrease the blood sugar level; this needs further studies.

**TABLE 5:** Retention time (RT), height and area of silymarin constituents and quercetin in standard solutions and optimum sample

Response	RT	Height	Area
Taxifolin (standard solution)	3.2	6.2	1,962.074
Silychristin (standard solution)	4.2	6.16	1,761.146
Silydianin (standard solution)	6.2	2.5	1,001.131
SilybininA (standard solution)	16.45	17.14	3,924.149
SilybininB (standard solution)	16.57	16.10	4,001.162
IsosilybininA (standard solution)	17.41	7.54	333.712
IsosilybininB (standard solution)	17.98	13.2	1,798.921
Taxifolin (optimum sample)	7.54	0.7	1,025.537
Silychristin (optimum sample)	8.98	0.5	1,134.417
Silydianin (optimum sample)	12.16	0.3	579.208
SilybininA (optimum sample)	14.342	2.9	8,204.299
SilybininB (optimum sample)	21.32	2.7	4,102.149
IsosilybininA (optimum sample)	22.84	0.4	820.219
IsosilybininB (optimum sample)	24.42	0.3	991.349
Quercetin (standard solution)	16.233	29.144	111.34
Quercetin (optimum sample)	16.300	55.4	36.78

**TABLE 6:** Theoretical versus practical values of the optimal sample

Value	Theoretical	Experimental
Density (g/cm^3^)	0.56	0.61 ± 0.03
Volume (cm^3^)	82.93	73.44 ± 2.02
Moisture % (Day 1)	19.07	22.20 ± 1.16
Moisture % (Day 30)	16.17	8.04 ± 0.45
Protein (%db)	12.46	12.14 ± 0.21
Ash (%db)	1.16	1.64 ± 0.22
Inhibition of DPPH %	40.63	31.31 ± 2.32
Hardness (g) (Day 1)	465.92	354 ± 31.02
Hardness (g) (Day 30)	871.22	1,203 ± 152.73
Springiness (mm) (Day 1)	7.48	7.71 ± 0.51
Springiness (mm) (Day 30)	6.08	6.21 ± 0.42
Cohesiveness (Day 1)	0.45	0.41 ± 0.06
Cohesiveness (Day 30)	0.33	0.40 ± 0.15
Paunch (g) (Day 1)	84.46	72.94 ± 1.21
Paunch (g) (Day 30)	208.90	173.89 ± 5.02
a*	−2.43	−2.36 ± 0.19
b*	7.78	7.42 ± 1.20
L*	16.81	15.09 ± 0.23
*∆E*	24.76	36.02 ± 2.87
SI	7.85	8.19 ± 0.03
BI	81.53	70.12 ± 4.98
H	−80.38	−66.27 ± 6.23
WI	16.43	15.88 ± 1.22
Silymarin (mg)	886.70	880.14 ± 0.14
Quercetin (mg)	62.90	63.01 ± 0.21
Texture	3.72	3.70±0.20
Flavor	3.78	3.28 ± 0.16
Color	3.08	3.44 ± 0.16
Odor	3.71	4.51 ± 0.16
Overall liking	3.720	3.70 ± 0.22
Antimicrobial	2.14	2.03 ± 0.32

## 4 CONCLUSION

Nettle and MT seed, as herbs with several medicinal benefits, can be used in the food industry as functional foods. The main composition of nettle leaf and MT seed is quercetin and silymarin, respectively. The combination of nettle and MT seeds has not side effects for human health. Certainly, while maintaining the positive health effects, it has very few side effects on the physical and chemical properties of the cake. Among the treatments, 12.5% nettle and 12.5% thistle have the lowest hardness and the highest volume, moisture, springiness and cohesiveness, and organoleptic properties, including color, taste, flavor, and overall acceptability. Increase of nettle and MT seeds in the cake is directly associated with increasing the amount of ash, fat, and protein. In this regard, the highest amount of fat and protein was observed in 25% MT samples. In all treatments, the microbial and antioxidant properties of the cake are improved due to the presence of phenolic and alkaloid substances in nettle leaves and MT seeds. The sample with 13.65% nettle and 11.34% MT seed was identified by the software based on the highest scores of sensory tests and the lowest level of hardness, as the optimal sample with a desirability of 0.72, and physical and chemical tests were performed on it. The optimal sample contained 62.90 mg quercetin and 886.70 mg silymarin. The results demonstrated that approximately 10 optimal cakes could theoretically decrease the blood sugar level, which needs further studies. The amount of quercetin and silymarin in 2.5 g of nettle leaves and 2.5 g of MT seed was 0.44% w/w equal to 11 mg and 0.87% w/w equal to 11 mg, respectively. After heating, their amount was 11 mg and 19 mg, respectively. In other words, heat did not have considerable effect on the destruction of quercetin and silymarin, and the substances could resist the thermal processes of the food industry. It is recommended that nettle and MT seeds be used in various food industries, particularly in the cereal industry in order to have acceptable physical and chemical properties and to use its medicinal properties like lowering the blood sugar. In this respect, further studies are required.

## Data Availability

The data that support the findings of this study are available from the Shahrekord Azad University upon reasonable request.
